# Supplementary figures and images for: Opportunistic consumption of marine pelagic, terrestrial, and chemosynthetic organic matter by macrofauna on the Arctic shelf: a stable isotope approach
Source: PeerJ. 2023 Jun 29;11:e15595. doi: 10.7717/peerj.15595 (PMC10315133; doi:10.7717/peerj.15595)

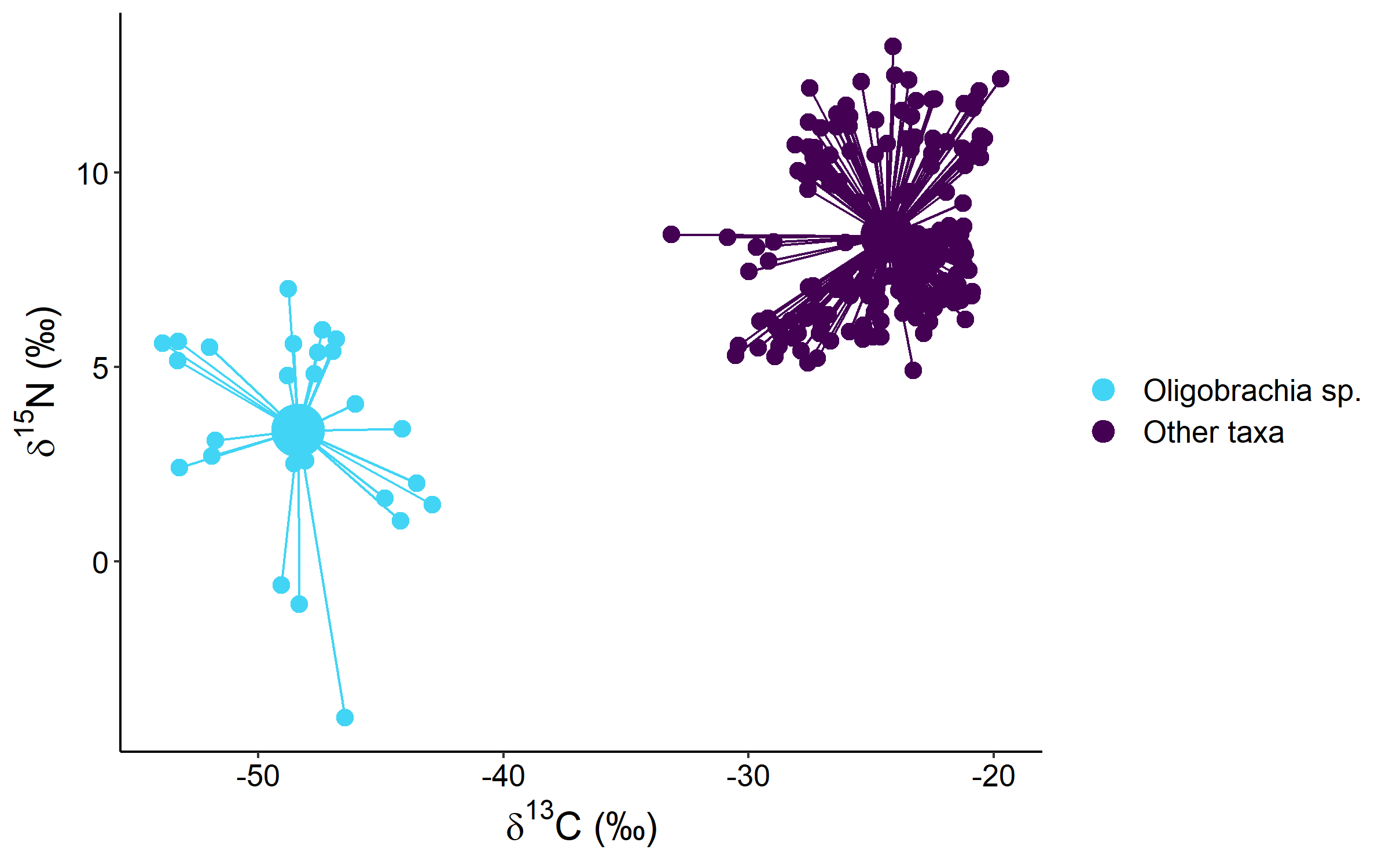

Supplement: Supplemental Information 1 — Group centroids (calculated as mean δ13C and δ15N values) are shown. [file peerj-11-15595-s001.png]
