# Supplementary material for: Opportunistic consumption of marine pelagic, terrestrial, and chemosynthetic organic matter by macrofauna on the Arctic shelf: a stable isotope approach
Source: PeerJ. 2023 Jun 29;11:e15595. doi: 10.7717/peerj.15595 (PMC10315133; doi:10.7717/peerj.15595)

**Supplemental Figure S2. Relationship between δ^13^C of methane and δ^13^C of Oligobrachia (mean ± sd) at several seep locations in the Arctic and North Atlantic**. Based on the data from the present study and Gebruk et al., 2003; Decker and Olu, 2012; Lee et al., 2019a; Åström et al., 2022; Kravchishina et al., 2021. HMMV = Haakon Mosby Mud Volcano.


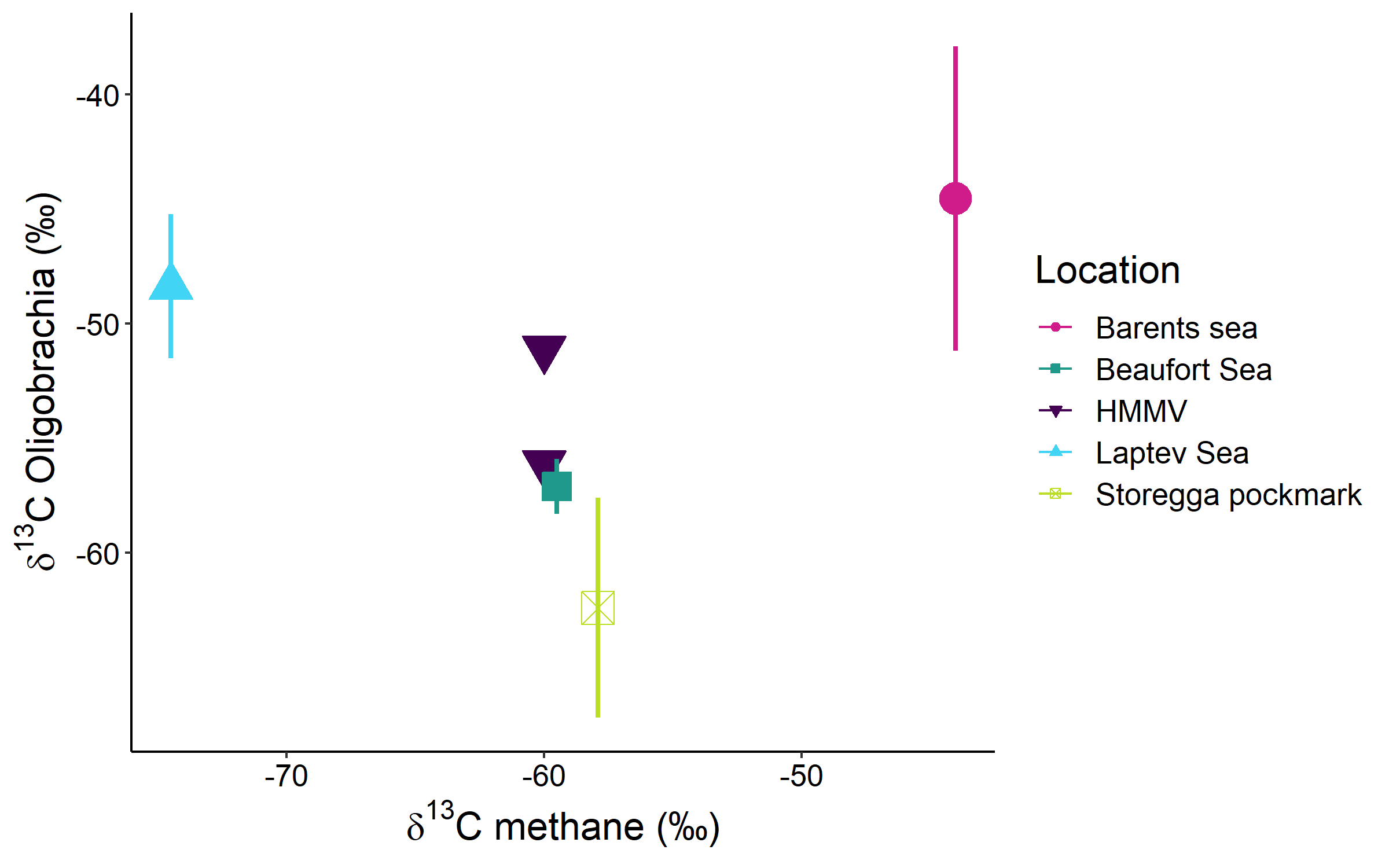

Supplement: Supplemental Information 2 — Based on the data from the present study and Gebruk et al., 2003; Decker & Olu, 2012; Lee et al., 2019a; Åström, Bluhm & Rasmussen, 2022; Kravchishina et al., 2021. HMMV, Haakon Mosby Mud Volcano. [file peerj-11-15595-s002.docx]
