# Supplementary material for: Opportunistic consumption of marine pelagic, terrestrial, and chemosynthetic organic matter by macrofauna on the Arctic shelf: a stable isotope approach
Source: PeerJ. 2023 Jun 29;11:e15595. doi: 10.7717/peerj.15595 (PMC10315133; doi:10.7717/peerj.15595)
Supplement: Supplemental Information 3 [file peerj-11-15595-s003.docx]

**Supplemental Table S1. δ^13^C, δ^15^N, and C/N values for macrofaunal samples.**

| **Habitat** | **Station** | **Species** | **δ^13^C vpdb** | **δ^15^N air** | **mass C/N** |
| --- | --- | --- | --- | --- | --- |
| Background | 6950 | *Ennucula tenuis* | -25.3 | 6.1 | 7.9 |
| Background | 6950 | *Macoma moesta* | -22.5 | 6.8 | 3.7 |
| Background | 6950 | *Macoma* sp. | -23.4 | 7.4 | 4.7 |
| Background | 6950 | *Maldane sarsi* | -23.6 | 10.9 | 4.6 |
| Background | 6950 | *Maldane sarsi* | -23.4 | 10.6 | 4.6 |
| Background | 6950 | *Maldane sarsi* | -23.2 | 11.8 | 4.1 |
| Background | 6950 | *Maldane sarsi* | -22.5 | 11.9 | 4.3 |
| Background | 6950 | *Maldane sarsi* | -23.2 | 10.9 | 5.0 |
| Background | 6950 | *Myriochele heeri* | -21.7 | 7.1 | 6.0 |
| Background | 6950 | Nephtyidae gen. sp. | -25.4 | 12.3 | 3.7 |
| Background | 6950 | *Nuculana pernula* | -23.0 | 6.7 | 3.7 |
| Background | 6950 | *Owenia polaris* | -21.3 | 8.4 | 4.8 |
| Background | 6950 | *Parathyasira dunbari* | -22.9 | 5.9 | 4.0 |
| Background | 6950 | *Pectinaria hyperborea* | -22.5 | 10.9 | 5.0 |
| Background | 6950 | *Pectinaria hyperborea* | -21.1 | 10.5 | 3.7 |
| Background | 6950 | *Periploma aleuticum* | -22.6 | 6.8 | 5.2 |
| Background | 6950 | *Portlandia arctica* | -21.3 | 6.8 | 3.7 |
| Background | 6950 | *Portlandia arctica* | -21.2 | 6.2 | 3.7 |
| Background | 6950 | *Sternapsis* sp. | -21.3 | 10.6 | 3.7 |
| Background | 6950 | *Sternapsis* sp. | -20.8 | 10.6 | 3.6 |
| Background | 6950 | *Thyasira* cf. *gouldii* | -23.2 | 8.0 | 4.0 |
| Background | 6950 | *Yoldiella lenticula* | -21.5 | 6.7 | 5.1 |
| Background | 6950 | *Yoldiella lenticula* | -23.7 | 6.4 | 4.6 |
| Background | 6950 | *Yoldiella lenticula* | -23.6 | 6.5 | 4.5 |
| Delta | 6976 | Nephtyidae gen. sp. | -27.3 | 10.6 | 4.4 |
| Delta | 6976 | Nephtyidae gen. sp. | -27.6 | 11.3 | 4.2 |
| Delta | 6976 | Nephtyidae gen. sp. | -24.8 | 11.4 | 3.6 |
| Delta | 6976 | *Thyasira* cf. *gouldii* | -28.9 | 6.0 | 4.5 |
| Delta | 6976 | *Thyasira* cf. *gouldii* | -27.9 | 5.4 | 4.0 |
| Delta | 6976 | *Thyasira* cf. *gouldii* | -28.3 | 6.2 | 4.4 |
| Delta | 6977 | *Macoma calcarea* | -28.7 | 5.9 | 3.7 |
| Delta | 6977 | *Macoma calcarea* | -28.7 | 5.5 | 3.7 |
| Delta | 6977 | *Macoma moesta* | -29.5 | 6.2 | 3.8 |
| Delta | 6977 | *Macoma moesta* | -29.2 | 6.3 | 3.9 |
| Delta | 6977 | *Pectinaria hyperborea* | -26.4 | 9.7 | 3.8 |
| Delta | 6977 | *Pectinaria hyperborea* | -27.6 | 10.7 | 4.5 |
| Delta | 6977 | *Pectinaria hyperborea* | -27.4 | 10.0 | 4.8 |
| Delta | 6977 | *Portlandia arctica* | -30.4 | 5.6 | 3.8 |
| Delta | 6977 | *Portlandia arctica* | -28.0 | 5.9 | 4.0 |
| Delta | 6977 | *Portlandia arctica* | -29.6 | 5.5 | 3.9 |
| Delta | 6977 | *Portlandia arctica* | -28.2 | 5.7 | 3.7 |
| Delta | 6977 | *Portlandia arctica* | -28.9 | 5.3 | 3.6 |
| Delta | 6977 | *Sternapsis* sp. | -25.1 | 6.8 | 4.1 |
| Delta | 6977 | *Sternapsis* sp. | -26.1 | 7.2 | 5.1 |
| Delta | 6977 | *Sternapsis* sp. | -24.6 | 7.3 | 3.8 |
| Delta | 6977 | *Thyasira* cf. *gouldii* | -27.6 | 5.1 | 4.8 |
| Delta | 6977 | *Thyasira* cf. *gouldii* | -27.2 | 5.2 | 4.2 |
| Delta | 6977 | *Thyasira* cf. *gouldii* | -30.5 | 5.3 | 4.8 |
| Seep | 5625 | *Frigidoalvania* sp. | -27.4 | 7.1 | 4.5 |
| Seep | 5625 | *Frigidoalvania* sp. | -27.7 | 6.2 | 5.6 |
| Seep | 5625 | *Frigidoalvania* sp. | -27.2 | 6.4 | 4.9 |
| Seep | 5625 | *Frigidoalvania* sp. | -27.6 | 7.0 | 5.2 |
| Seep | 5625 | *Frigidoalvania* sp. | -27.3 | 6.9 | 4.6 |
| Seep | 5625 | *Frigidoalvania* sp. | -26.4 | 7.0 | 4.5 |
| Seep | 6939 | *Maldane sarsi* | -22.4 | 11.9 | 3.5 |
| Seep | 6939 | *Maldane sarsi* | -24.1 | 13.2 | 3.8 |
| Seep | 6939 | *Myriochele heeri* | -22.2 | 8.3 | 4.7 |
| Seep | 6939 | *Myriochele heeri* | -21.5 | 7.4 | 5.1 |
| Seep | 6939 | *Myriochele heeri* | -22.1 | 7.9 | 5.9 |
| Seep | 6939 | *Myriochele heeri* | -21.9 | 7.0 | 4.9 |
| Seep | 6939 | *Oligobrachia* sp. | -48.8 | 7.0 | 5.1 |
| Seep | 6939 | *Oligobrachia* sp. | -46.8 | 5.7 | 4.9 |
| Seep | 6939 | *Oligobrachia* sp. | -47.4 | 6.0 | 5.2 |
| Seep | 6939 | *Spiochaetopterus typicus* | -22.7 | 7.7 | 5.9 |
| Seep | 6939 | *Spiochaetopterus typicus* | -21.3 | 8.1 | 4.2 |
| Seep | 6947 | *Ennucula tenuis* | -25.2 | 5.8 | 6.3 |
| Seep | 6947 | *Maldane sarsi* | -26.4 | 11.5 | 4.1 |
| Seep | 6947 | *Maldane sarsi* | -26.7 | 10.5 | 4.0 |
| Seep | 6947 | *Maldane sarsi* | -26.7 | 9.7 | 3.9 |
| Seep | 6947 | *Maldane sarsi* | -26.0 | 11.7 | 3.8 |
| Seep | 6947 | *Maldane sarsi* | -26.1 | 11.6 | 3.9 |
| Seep | 6947 | *Montacuta spitzbergensis* | -29.2 | 7.7 | 3.9 |
| Seep | 6947 | *Myriochele heeri* | -23.1 | 7.6 | 5.6 |
| Seep | 6947 | *Myriochele heeri* | -23.1 | 6.9 | 5.4 |
| Seep | 6947 | *Myriochele heeri* | -23.5 | 6.7 | 5.2 |
| Seep | 6947 | Nephtyidae gen. sp. | -25.9 | 11.4 | 3.5 |
| Seep | 6947 | Nephtyidae gen. sp. | -26.2 | 11.4 | 3.6 |
| Seep | 6947 | Nephtyidae gen. sp. | -27.4 | 10.4 | 3.5 |
| Seep | 6947 | *Nuculana pernula* | -25.3 | 7.3 | 3.4 |
| Seep | 6947 | *Nuculana pernula* | -25.3 | 5.7 | 3.7 |
| Seep | 6947 | *Nuculana pernula* | -25.9 | 5.9 | 3.7 |
| Seep | 6947 | *Nuculana pernula* | -27.7 | 6.4 | 4.8 |
| Seep | 6947 | *Nuculana pernula* | -24.9 | 7.0 | 3.6 |
| Seep | 6947 | *Oligobrachia* sp. | -53.2 | 2.4 | 5.5 |
| Seep | 6947 | *Oligobrachia* sp. | -52.0 | 5.5 | 6.0 |
| Seep | 6947 | *Oligobrachia* sp. | -51.7 | 3.1 | 6.2 |
| Seep | 6947 | *Oligobrachia* sp. | -51.9 | 2.7 | 5.7 |
| Seep | 6947 | *Owenia polaris* | -21.6 | 8.3 | 5.3 |
| Seep | 6947 | *Owenia polaris* | -21.4 | 8.5 | 4.4 |
| Seep | 6947 | *Owenia polaris* | -21.2 | 8.6 | 4.6 |
| Seep | 6947 | *Pectinaria hyperborea* | -26.3 | 9.8 | 5.6 |
| Seep | 6947 | *Pectinaria hyperborea* | -25.9 | 11.2 | 4.6 |
| Seep | 6947 | *Pectinaria hyperborea* | -27.6 | 9.6 | 5.4 |
| Seep | 6947 | *Pectinaria hyperborea* | -28.1 | 10.7 | 5.0 |
| Seep | 6947 | *Pectinaria hyperborea* | -29.0 | 8.2 | 3.6 |
| Seep | 6947 | *Pectinaria hyperborea* | -26.8 | 10.0 | 3.9 |
| Seep | 6947 | *Portlandia arctica* | -24.6 | 5.8 | 5.5 |
| Seep | 6947 | *Portlandia arctica* | -25.2 | 5.9 | 3.6 |
| Seep | 6947 | *Portlandia arctica* | -23.6 | 6.8 | 4.7 |
| Seep | 6947 | *Thyasira* cf. *gouldii* | -30.0 | 7.5 | 4.4 |
| Seep | 6947 | *Yoldia hyperborea* | -24.9 | 6.4 | 3.7 |
| Seep | 6947 | *Yoldiella lenticula* | -26.7 | 5.7 | 4.0 |
| Seep | 6947 | *Yoldiella lenticula* | -27.0 | 6.1 | 4.8 |
| Seep | 6947 | *Yoldiella lenticula* | -27.1 | 5.9 | 3.8 |
| Seep | 6947 | *Yoldiella solidula* | -25.2 | 7.6 | 4.4 |
| Seep | 6952 | *Macoma calcarea* | -25.9 | 6.8 | 3.6 |
| Seep | 6952 | *Maldane sarsi* | -25.9 | 10.6 | 3.7 |
| Seep | 6952 | *Maldane sarsi* | -27.1 | 11.2 | 4.8 |
| Seep | 6952 | *Maldane sarsi* | -27.4 | 10.1 | 3.5 |
| Seep | 6952 | *Myriochele heeri* | -23.0 | 8.0 | 6.3 |
| Seep | 6952 | *Myriochele heeri* | -22.2 | 8.5 | 7.8 |
| Seep | 6952 | *Myriochele heeri* | -21.8 | 8.6 | 7.6 |
| Seep | 6952 | *Myriochele heeri* | -22.2 | 8.0 | 7.2 |
| Seep | 6952 | *Myriochele heeri* | -23.1 | 6.6 | 5.6 |
| Seep | 6952 | *Myriochele heeri* | -23.1 | 7.0 | 6.4 |
| Seep | 6952 | *Myriochele heeri* | -22.7 | 7.4 | 5.8 |
| Seep | 6952 | *Myriochele heeri* | -22.7 | 7.4 | 5.8 |
| Seep | 6952 | Nephtyidae gen. sp. | -23.8 | 11.6 | 3.6 |
| Seep | 6952 | Nephtyidae gen. sp. | -23.4 | 11.4 | 3.7 |
| Seep | 6952 | Nephtyidae gen. sp. | -24.0 | 12.5 | 3.6 |
| Seep | 6952 | Nephtyidae gen. sp. | -27.5 | 12.2 | 3.7 |
| Seep | 6952 | Nephtyidae gen. sp. | -23.5 | 12.4 | 3.6 |
| Seep | 6952 | *Oligobrachia* sp. | -46.0 | 4.1 | 4.8 |
| Seep | 6952 | *Oligobrachia* sp. | -48.3 | -1.1 | 5.1 |
| Seep | 6952 | *Oligobrachia* sp. | -53.9 | 5.6 | 4.7 |
| Seep | 6952 | *Oligobrachia* sp. | -53.3 | 5.2 | 4.2 |
| Seep | 6952 | *Oligobrachia* sp. | -47.6 | 5.4 | 4.3 |
| Seep | 6952 | *Oligobrachia* sp. | -43.5 | 2.0 | 4.6 |
| Seep | 6952 | *Oligobrachia* sp. | -47.0 | 5.4 | 5.1 |
| Seep | 6952 | *Oligobrachia* sp. | -47.7 | 4.8 | 5.6 |
| Seep | 6952 | *Oligobrachia* sp. | -48.1 | 2.6 | 4.0 |
| Seep | 6952 | *Oligobrachia* sp. | -42.9 | 1.5 | 4.6 |
| Seep | 6952 | *Oligobrachia* sp. | -53.2 | 5.7 | 5.3 |
| Seep | 6952 | *Owenia polaris* | -21.7 | 8.4 | 4.6 |
| Seep | 6952 | *Owenia polaris* | -20.9 | 6.9 | 7.0 |
| Seep | 6952 | *Owenia polaris* | -22.3 | 8.3 | 4.3 |
| Seep | 6952 | *Owenia polaris* | -22.2 | 8.4 | 4.1 |
| Seep | 6952 | *Owenia polaris* | -20.9 | 6.8 | 6.6 |
| Seep | 6952 | *Owenia polaris* | -21.6 | 7.9 | 5.4 |
| Seep | 6952 | *Periploma aleuticum* | -21.9 | 9.5 | 3.5 |
| Seep | 6952 | *Portlandia arctica* | -21.5 | 6.8 | 3.8 |
| Seep | 6952 | *Portlandia arctica* | -21.4 | 6.7 | 3.7 |
| Seep | 6952 | *Portlandia arctica* | -21.6 | 6.6 | 3.7 |
| Seep | 6952 | *Sternapsis* sp. | -33.2 | 8.4 | 3.9 |
| Seep | 6952 | *Sternapsis* sp. | -21.2 | 11.8 | 4.0 |
| Seep | 6952 | *Sternapsis* sp. | -23.5 | 9.5 | 3.5 |
| Seep | 6952 | *Thracia myopsis* | -30.9 | 8.3 | 4.1 |
| Seep | 6952 | *Thracia myopsis* | -29.7 | 8.1 | 4.3 |
| Seep | 6952 | *Thyasira* cf. *gouldii* | -23.4 | 6.8 | 3.9 |
| Seep | 6952 | *Yoldiella lenticula* | -24.2 | 7.3 | 6.2 |
| Seep | 6953 | *Maldane sarsi* | -27.7 | 10.0 | 4.0 |
| Seep | 6953 | *Maldane sarsi* | -28.0 | 10.1 | 3.8 |
| Seep | 6953 | *Maldane sarsi* | -26.4 | 11.2 | 3.8 |
| Seep | 6953 | *Maldane sarsi* | -24.3 | 10.7 | 3.9 |
| Seep | 6953 | *Maldane sarsi* | -26.0 | 11.4 | 4.2 |
| Seep | 6953 | *Maldane sarsi* | -26.9 | 10.4 | 3.7 |
| Seep | 6953 | *Myriochele heeri* | -22.7 | 7.8 | 5.1 |
| Seep | 6953 | *Myriochele heeri* | -23.2 | 6.3 | 5.4 |
| Seep | 6953 | *Myriochele heeri* | -22.8 | 6.8 | 4.9 |
| Seep | 6953 | *Myriochele heeri* | -22.3 | 7.8 | 5.7 |
| Seep | 6953 | *Myriochele heeri* | -23.0 | 8.0 | 5.9 |
| Seep | 6953 | Nephtyidae gen. sp. | -23.2 | 10.9 | 3.7 |
| Seep | 6953 | *Oligobrachia* sp. | -46.5 | -4.0 | 5.0 |
| Seep | 6953 | *Oligobrachia* sp. | -48.5 | 2.5 | 7.9 |
| Seep | 6953 | *Oligobrachia* sp. | -48.2 | 3.2 | 6.3 |
| Seep | 6953 | *Oligobrachia* sp. | -44.1 | 3.4 | 3.8 |
| Seep | 6953 | *Pectinaria hyperborea* | -22.6 | 10.2 | 6.9 |
| Seep | 6953 | *Pectinaria hyperborea* | -24.8 | 10.5 | 4.4 |
| Seep | 6953 | *Sternapsis* sp. | -23.1 | 8.4 | 4.2 |
| Seep | 6953 | *Sternapsis* sp. | -23.8 | 9.4 | 4.1 |
| Seep | 6953 | *Sternapsis* sp. | -25.5 | 9.2 | 3.6 |
| Seep | 6953 | *Thyasira* cf. *gouldii* | -26.0 | 8.2 | 4.0 |
| Seep | 6953 | *Thyasira* cf. *gouldii* | -25.4 | 7.0 | 4.1 |
| Seep | 6953 | *Yoldiella lenticula* | -26.8 | 6.3 | 4.1 |
| Seep | 6953 | *Yoldiella solidula* | -24.3 | 8.0 | 6.0 |
| Seep | 6953 | *Yoldiella solidula* | -25.1 | 7.8 | 6.9 |
| Seep | 6992 | *Macoma calcarea* | -22.1 | 7.2 | 3.7 |
| Seep | 6992 | *Macoma* sp. | -23.8 | 7.0 | 4.2 |
| Seep | 6992 | *Macoma* sp. | -22.4 | 6.5 | 4.0 |
| Seep | 6992 | *Macoma* torelli | -24.6 | 6.2 | 5.5 |
| Seep | 6992 | *Maldane sarsi* | -22.5 | 10.8 | 3.8 |
| Seep | 6992 | *Maldane sarsi* | -21.9 | 10.8 | 3.6 |
| Seep | 6992 | *Maldane sarsi* | -22.5 | 10.5 | 3.9 |
| Seep | 6992 | *Myriochele heeri* | -21.2 | 8.1 | 5.3 |
| Seep | 6992 | *Myriochele heeri* | -21.5 | 8.2 | 5.5 |
| Seep | 6992 | *Myriochele heeri* | -21.2 | 7.9 | 6.1 |
| Seep | 6992 | *Myriochele heeri* | -21.3 | 9.2 | 4.9 |
| Seep | 6992 | *Myriochele heeri* | -21.5 | 7.1 | 6.7 |
| Seep | 6992 | *Myriochele heeri* | -21.2 | 7.6 | 4.9 |
| Seep | 6992 | *Myriochele heeri* | -21.0 | 7.5 | 5.2 |
| Seep | 6992 | *Myriochele heeri* | -21.8 | 8.3 | 6.3 |
| Seep | 6992 | Nephtyidae gen. sp. | -20.8 | 11.9 | 3.9 |
| Seep | 6992 | Nephtyidae gen. sp. | -19.7 | 12.4 | 3.7 |
| Seep | 6992 | Nephtyidae gen. sp. | -20.6 | 10.9 | 3.7 |
| Seep | 6992 | Nephtyidae gen. sp. | -20.6 | 12.1 | 3.7 |
| Seep | 6992 | Nephtyidae gen. sp. | -20.9 | 11.6 | 3.7 |
| Seep | 6992 | *Nuculana pernula* | -22.6 | 6.6 | 3.6 |
| Seep | 6992 | *Nuculana pernula* | -22.4 | 6.8 | 3.6 |
| Seep | 6992 | *Nuculana pernula* | -22.6 | 6.2 | 3.4 |
| Seep | 6992 | *Nuculana pernula* | -22.1 | 6.7 | 3.7 |
| Seep | 6992 | *Nuculana pernula* | -23.0 | 6.6 | 3.5 |
| Seep | 6992 | *Nuculana pernula* | -24.9 | 5.8 | 9.6 |
| Seep | 6992 | *Nuculana pernula* | -23.9 | 7.0 | 5.3 |
| Seep | 6992 | *Oligobrachia* sp. | -48.5 | 5.6 | 4.3 |
| Seep | 6992 | *Oligobrachia* sp. | -49.0 | -0.6 | 5.8 |
| Seep | 6992 | *Oligobrachia* sp. | -48.8 | 4.8 | 5.2 |
| Seep | 6992 | *Oligobrachia* sp. | -44.2 | 1.0 | 4.4 |
| Seep | 6992 | *Oligobrachia* sp. | -44.8 | 1.6 | 5.0 |
| Seep | 6992 | *Parathyasira dunbari* | -23.3 | 4.9 | 4.1 |
| Seep | 6992 | *Sternapsis* sp. | -20.6 | 10.7 | 3.9 |
| Seep | 6992 | *Sternapsis* sp. | -20.6 | 10.4 | 3.8 |
| Seep | 6992 | *Sternapsis* sp. | -20.5 | 10.9 | 3.9 |
| Seep | 6992 | *Sternapsis* sp. | -21.2 | 10.2 | 4.3 |
| Seep | 6992 | *Sternapsis* sp. | -20.4 | 10.9 | 3.8 |
| Seep | 6992 | *Sternapsis* sp. | -20.6 | 10.7 | 3.7 |
| Seep | 6992 | *Thyasira* cf. *gouldii* | -23.0 | 7.5 | 4.2 |
| Seep | 6992 | *Yoldiella lenticula* | -23.7 | 7.4 | 5.2 |
| Seep | 6992 | *Yoldiella lenticula* | -24.6 | 6.7 | 5.2 |
