# Supplementary material for: Opportunistic consumption of marine pelagic, terrestrial, and chemosynthetic organic matter by macrofauna on the Arctic shelf: a stable isotope approach
Source: PeerJ. 2023 Jun 29;11:e15595. doi: 10.7717/peerj.15595 (PMC10315133; doi:10.7717/peerj.15595)
Supplement: Supplemental Information 4 [file peerj-11-15595-s004.docx]

**Supplemental Table S2. Data on the sampling stations.**

| **Cruise** | **Date** | **Station** | **Longitude °E** | **Latitude °N** | **Depth, m** | **Habitat** |
| --- | --- | --- | --- | --- | --- | --- |
| AMK-82 | 09.10.2020 | 6950 | 127.02 | 76.88 | 69 | Background |
| AMK-82 | 18.10.2020 | 6977 | 130.36 | 73.11 | 22.5 | Delta |
| AMK-82 | 18.10.2020 | 6976 | 130.37 | 73.11 | 25 | Delta |
| AMK-82 | 07.10.2020 | 6939 | 122.10 | 77.28 | 293.5 | Seep |
| AMK-82 | 09.10.2020 | 6952 | 127.79 | 76.89 | 64 | Seep |
| AMK-82 | 09.10.2020 | 6953 | 127.82 | 76.90 | 65 | Seep |
| AMK-82 | 21.10.2020 | 6992 | 125.43 | 76.39 | 51.5 | Seep |
| AMK-82 | 08.10.2020 | 6947 | 125.83 | 76.78 | 72 | Seep |
| AMK-69 | 16.09.2017 | 5625 | 125.82 | 76.77 | 70.8 | Seep |
