# Supplementary material for: Opportunistic consumption of marine pelagic, terrestrial, and chemosynthetic organic matter by macrofauna on the Arctic shelf: a stable isotope approach
Source: PeerJ. 2023 Jun 29;11:e15595. doi: 10.7717/peerj.15595 (PMC10315133; doi:10.7717/peerj.15595)
Supplement: Supplemental Information 5 — Significant pair-wise comparisons are in bold. [file peerj-11-15595-s005.docx]

**Supplementary Table S3. Results of Kruskal-Wallis test and Dunn’s test for differences in the range of δ^13^C values at different sampling stations (excluding *Oligobrachia* sp.).** Significant pair-wise comparisons are in bold.

| **Kruskal-Wallis** | **Dunn's Test** | | | |
| --- | --- | --- | --- | --- |
|  | **Z** | **P** | **P.adjusted** | **Comparisons** |
| H = 95.8, df = 6, p < 0.001 | **3.5** | **< 0.001** | **0.003** | **6939 - 6947** |
|  | 0.5 | 0.298 | 0.597 | 6939 - 6950 |
|  | **-4.4** | **< 0.001** | **< 0.001** | **6947 - 6950** |
|  | 1.4 | 0.078 | 0.388 | 6939 - 6952 |
|  | **-3.4** | **< 0.001** | **0.005** | **6947 - 6952** |
|  | 1.3 | 0.099 | 0.397 | 6950 - 6952 |
|  | 2.6 | 0.005 | 0.046 | 6939 - 6953 |
|  | -1.1 | 0.130 | 0.390 | 6947 - 6953 |
|  | **2.9** | **0.002** | **0.021** | **6950 - 6953** |
|  | 1.8 | 0.033 | 0.232 | 6952 - 6953 |
|  | **4.9** | **< 0.001** | **< 0.001** | **6939 - 6976/6977** |
|  | 2.4 | 0.009 | 0.074 | 6947 - 6976/6977 |
|  | **6.1** | **< 0.001** | **< 0.001** | **6950 - 6976/6977** |
|  | **5.4** | **< 0.001** | **< 0.001** | **6952 - 6976/6977** |
|  | **3.1** | **0.001** | **0.010** | **6953 - 6976/6977** |
|  | -0.4 | 0.340 | 0.340 | 6939 - 6992 |
|  | **-6.5** | **< 0.001** | **< 0.001** | **6947 - 6992** |
|  | -1.4 | 0.075 | 0.450 | 6950 - 6992 |
|  | **-3.0** | **0.001** | **0.013** | **6952 - 6992** |
|  | **-4.5** | **< 0.001** | **< 0.001** | **6953 - 6992** |
|  | **-8.2** | **< 0.001** | **< 0.001** | **6976/6977 - 6992** |
